# Was the steppe bison a grazing beast in Pleistocene landscapes?

**DOI:** 10.1098/rsos.240317

**Published:** 2024-08-14

**Authors:** Emilia Hofman-Kamińska, Gildas Merceron, Hervé Bocherens, Gennady G. Boeskorov, Oleksandra O. Krotova, Albert V. Protopopov, Andrei V. Shpansky, Rafał Kowalczyk

**Affiliations:** ^1^ Mammal Research Institute, Polish Academy of Sciences, ul. Stoczek 1, 17-230 Białowieża, Poland; ^2^ PALEVOPRIM lab, UMR 7262 CNRS & University of Poitiers, Bat. B35—TSA-51106, 86073 Poitiers Cedex 9, France; ^3^ Fachbereich Geowissenschaften, Forschungsbereich Paläobiologie, Universität Tübingen, Hölderlinstr. 12, Germany; ^4^ Senckenberg Centre for Human Evolution and Palaeoecology (HEP), Universität Tübingen, Hölderlinstr. 12, Germany; ^5^ The Geological Museum of the Diamond and Precious Metals Geology Institute, Siberian Branch, Russian Academy of Sciences, Lenina 39, Yakutsk 677007, Russia; ^6^ Mammoth Fauna Research Department, Academy of Sciences of the Republic of Sakha (Yakutia), Lenina Prospekt 33, Yakutsk, Republic of Sakha (Yakutia) 677007, Russia; ^7^ Stone Age Archaeology Department, Institute of Archaeology of the National Academy of Sciences of Ukraine, 12 Volodymyr Ivasyuk avenue, Kyiv 04210, Ukraine; ^8^ Department of Paleontology and Historical Geology, Tomsk State University, Lenina prospekt 36, Tomsk 634050, Russia

**Keywords:** *Bison priscus*, Bb1 bison, dental microwear textures, diet, habitat use, vegetation modelling

## Abstract

The history and palaeoecology of the steppe bison (*Bison priscus*) remain incompletely understood despite its widespread distribution. Using dental microwear textural analysis (DMTA) and vegetation modelling, we reconstructed the diet and assessed the habitat of steppe bison inhabiting Eurasia and Alaska since the Middle Pleistocene. During the Late Pleistocene, steppe bison occupied a variety of biome types: from the mosaic of temperate summergreen forest and steppe/temperate grassland (Serbia) to the tundra biomes (Siberia and Alaska). Despite the differences in the identified biome types, the diet of steppe bison did not differ significantly among populations in Eurasia. DMTA classified it as a mixed forager in all populations studied. The DMTA of Bb1 bison—a recently identified genetically extinct sister-clade of *Bison bonasus*—was typical of a highly grazing bovid species and differed from all *B. priscus* populations. The results of the study temper the common perception that steppe bison were grazers in steppe habitats. The dietary plasticity of the steppe bison was lower when compared with modern European bison and may have played an important role in its extinction, even in the stable tundra biome of eastern Siberia, where it has survived the longest in all of Eurasia.

## 1. Introduction

Among modern and Late Pleistocene mammals, large-bodied herbivores had the highest proportion of species that were extinct or at high risk of extinction [1]. A recent multi-species modelling study specified that the most important driver of extinction risk for Pleistocene megafauna in Eurasia was first habitat fragmentation, then body mass (about 39% importance), landscape spatial structure (about 38% importance) followed by dietary preferences (about 20%) [2]. It follows that large-bodied, herbivorous mammals with narrow dietary specializations (strict grazers or browsers) whose habitats were severely fragmented were most vulnerable to extinction.

Steppe bison (*Bison priscus* Bojanus, 1825) was one of the most characteristic megafauna species of the Mid- and Late Pleistocene landscapes of the northern hemisphere, surviving regionally into the Holocene [3–5]. The youngest known steppe bison in Europe went extinct in France at least 17 650 ± 670 cal. years BP [6]. They were extinct in most of Siberia by about 15 000−11 600 years BP [7]. However, some populations survived in the south of Western Siberia and Yakutia until the early Holocene (10 700–9800 cal. years BP) [4,8], and even up to the Middle Holocene around 5452 cal. years BP in Yukon, North America [5]. Bone remains of this species have been identified from the Iberian Peninsula, through central and eastern Europe [9,10] into Western Siberia [11], Yakutia and across Beringia [12] to North America [13]. However, despite its widespread latitudinal distribution covering a wide gradient of vegetation during the Late Pleistocene [5,14–16], the palaeoecological knowledge of the steppe bison remains fragmentary. This is notably due to a combination of low numbers of specimens studied, the lack of accurate age determination and the potential confusion with aurochs (*Bos primigenius* Bojanus, 1825) [17].

Bison, including steppe bison, are considered grazing specialists due to their morpho-physiological adaptations [18,19]. This narrow dietary niche may have made them less adaptable to environmental changes during the Pleistocene–Holocene transition. Dental microwear analysis of steppe bison from Western Europe: La Berbie in France [20] as well as the stable nitrogen isotope analysis of *B. priscus* from sites in Belgium and France, all dated between 45 000 and 38 000 cal. years BP [21,22], identified it as a grazer. However, a two-dimensional dental microwear study showed that *B. priscus* from Germany was a mixed feeder or even a browser during the Eemian interglacial MIS-5e (130 000 years BP) as well as in the Oerel interstadial MIS-3 (58 000–54 000 years BP), while the Late Pleistocene steppe bison from Britain has been recognized as a mixed feeder or a grazer [23]. Dietary studies of Late Pleistocene/Holocene *B. priscus* from several sites in North America have shown a wide range of tooth microwear patterns, from pure grazing to mixed feeding to browsing [24]. Therefore, the absolute statement that the steppe bison was a purely grazing specialist is unfounded. On the other hand, the above-mentioned studies were conducted over different time periods and used different dietary analysis methods, making it difficult to determine patterns of dietary variation and plasticity across time and space.

Recent genetic studies have shown that Europe was inhabited not only by steppe bison in the Late Pleistocene, but also by two clades of *Bison bonasus*: Bb1, also called Clade X, which went extinct in the early Holocene; and Bb2, the clade that survived to the present [25]. Teeth from Bb1 bison from Amvrosiivka (Ukraine) were analysed previously as steppe bison (*B. priscus*) in the context of its migratory behaviour [26]. However, genetic analysis of several teeth from Amvrosiivka (Palaeolithic bison kill site with bone bed covered area up to 300 m^2^) [26] has revealed that these specimens belong to Bb1 bison [25]. A previous study based on stable carbon isotope analysis showed that Bb1 from this site foraged on C3 plants from open steppe/grassland with probably incorporation of lichens in winter [26].

Dental microwear analysis relates to the relationship between tooth wear patterns and the diet component of the species [27]. Focusing on ungulates, variations in tooth surface texture depend on the relative proportions of the amount of silica-bearing monocots and lignin-enriched dicots. These characteristics vary depending on the tissue type (leaves, blades, twigs, bark) and ontogenic development of the consumed plant [28–30]. Although grit ingested during foraging in low vegetation layers could alter the enamel surface [31,32], *in vivo* controlled feeding experiments on sheep with fine dust-laden diets simulating high levels of airborne fine dust did not overwhelm the dietary signal of the dental microwear textures [30]. Tooth microwear reflects the last few weeks of an animal’s foraging behaviour before its death [33,34]. It has even been shown that seasonal variations in diet can lead to differences in microwear patterns between individuals of the same population that died at different seasons of the year [35–37]. In order to capture a representation of the diet from all seasons of the year on the teeth, we analysed population represented by at least 10 individuals [38,39]. Dental microwear texture analysis (DMTA) has already been used to study the diet of many extant and extinct mammalian species [40–42], but never before to analyse the diet of the Eurasian steppe bison.

Investigating dietary plasticity as a mechanism for adaptation to climate–environment changes in the widespread steppe bison may help us understand the reasons for the megafaunal extinctions in the past. The aim of our study is to determine the dietary plasticity of steppe bison dispersed over large geographic (from Europe through Asia to Alaska) and temporal (from Middle to Late Pleistocene) scales.

Specific questions addressed in this study were: (i) Did the diet of the steppe bison vary across a gradient of vegetation/biome types? (ii) Was the steppe bison an obligate grazer with a narrow dietary spectrum, making it potentially less adaptable to environmental change at the Pleistocene–Holocene transition? (iii) Was the diet of *B. priscus* similar to that of its extinct relative Bb1? To answer our questions, we used dental microwear texture as a dietary proxy and pollen-based reconstruction of vegetation cover to identify biome types and proportions of each vegetation type at every study site individually [43].

## 2. Material and methods

### 2.1. Teeth origin and radiocarbon dating

**Figure 1.**
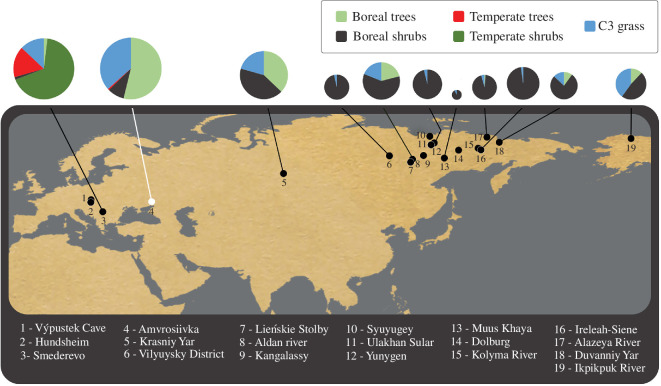
Location of specimens of the steppe bison (*B. priscus*)—black circles and Bb1 bison—white circle. For a detailed description of the study sites, see electronic supplementary material, S2. Circles indicate mean leaf area index (LAI) of reconstructed vegetation types for each study site. The size of the circle is proportional to the number of samples in the site. The age and vegetation reconstruction of individual samples are shown in electronic supplementary material, S1 and S4.1.

The material used in this study was collected between 2013 and 2017 from archaeological excavations and museum collections of the Institute of Palaeontology Department of Palaeontology University of Vienna and the Natural History Museum Vienna Department of Geology & Palaeontology Collection (Austria), the Natural History Museum of Belgrade (Serbia), the Institute of Archaeology Ukrainian Academy of Sciences Department of Stone Age (Ukraine) and the Mammoth Fauna Research Department, Academy of Sciences of the Republic of Sakha; the Geological Museum of the Institute of Geology of Diamonds and Precious Metals, Siberian Division Russian Academy of Sciences; the Museum of Mammoth of the Research Institute of Applied Ecology of the North, North-Eastern Federal University named after M. K. Ammosov in Yakutsk; the Sightseeing Museum and the Paleontology Museum of Tomsk State University (Russia). Material was originated from localities ranging from Central Europe through Western Siberia to Eastern Siberia in comparison with published steppe bison data from Alaska [44] in a gradient of different vegetation biomes [43] (figure 1; electronic supplementary material, S1). For a detailed description of the study sites, see electronic supplementary material, S2. We analysed specimens well identified to species level (teeth associated with preserved cranial/mandibular bones) from the early Middle (800–400 ka years BP) to the Late Pleistocene (>50 000–18 500 cal. years BP). Of 141 steppe bison specimens with preserved teeth, 46 were unsuitable for microwear analysis due to postmortem damage to the enamel surface and were therefore discarded from the analysis. For analysis, we used 95 original tooth moulds from Eurasian steppe bison and 14 scans of Alaskan *B. priscus* teeth [44] (electronic supplementary material, S1). For comparison with *B. priscus*, we additionally used 48 original tooth moulds from the specimens identified as Bb1 bison from Amvrosiivka (Ukraine), available at the Institute of Archaeology in Kyiv (Ukraine) (figure 1) and previously recognized as *B. priscus*. A total of 157 *Bison* tooth moulds were analysed (electronic supplementary material, S1).

Bone samples for the radiocarbon dating were taken from the same specimen as the tooth moulds for microwear analysis. Bone collagen was extracted at the University of Tübingen (Germany) and ^14^C radiocarbon dated (AMS) at the Laboratory of Ion Beam Physics, Eidgenössische Technische Hochschule (ETH) Zürich, Switzerland. Thirty-one new radiocarbon dates of *B. priscus* bones and one date of Bb1 bison were performed for this study (all with ETH laboratory numbers), and additionally, two age determinations were available from museum information (electronic supplementary material, S1). In addition, three ^14^C radiocarbon dates of *B. priscus* teeth from Alaska (Ikpikpuk) [44] were found in the published literature [45,46]. Of the 37 radiocarbon-dated specimens, we can calibrate the age of the 20 samples to perform a vegetation reconstruction. The other 16 specimens had infinite dates and vegetation reconstruction for 1 specimen with a radiocarbon date was not possible (electronic supplementary material, S1). Radiocarbon dates were calibrated using the IntCal20 calibration curve in OxCal v. 4.4 [47].

### 2.2. Moulding and dental microwear textural analysis

The molars were cleaned with acetone to remove potential adhesives, dust and other preservatives prior to moulding. The moulds of mostly maxillary or mandibular second molars were prepared with a polyvinylsiloxane elastomer (Regular Body President, ref. 6015-ISO 4823, medium consistency, polyvinylsiloxane addition type; Coltene Whaledent) [48]. Three-dimensional scans (333 µm × 251 µm area) of the moulds were made at the Paleoprim laboratory, CNRS and University of Poitiers, France. Protoconid distolabial facet of lower molars and protocone facets of upper molars were scanned with TRIDENT, a white light scanning confocal microscope (DCM8, Leica Microsystems) with a 100× objective (numerical aperture = 0.90; working distance = 0.9 mm) [30]. We avoided using different dental facets (protoconid with paracone on maxillary molars) as they have different functions during mastication and thus significant differences in dental microwear textures [39]. The lateral resolution is 0.129 μm and the vertical spacing is 0.002 μm. An area of 200 × 200 µm^2^ was cropped from the original scan and saved as a .Plµ file [49]. Since the original scans of Alaskan *B. priscus* teeth were analysed using four areas (100 × 140 µm^2^) extracted from 204 × 276 µm^2^ scans [44], we obtained the original MNT. files from the authors and cut them to the same size as our 200 × 200 µm^2^ area. The obtained scans were analysed with LeicaMap (v. 8.2) software using a scale-sensitive fractal analysis (Surfract). The following texture surface parameters were used (electronic supplementary material, S1): (i) area-scale fractal complexity (*Asfc*)—highly pitted surfaces with features of varying sizes typically have high complexity; (ii) exact proportion length-scale anisotropy of relief (*epLsar*)—a measure of surface texture directionality—a surface dominated by parallel straight scratches is highly anisotropic; and (iii) heterogeneity of area-scale fractal complexity (*HAsfc_9_* and *HAsfc_81_*)—a measure of variation across a surface through 9 cells for *HAsfc_9_* and through 81 cells for *HAsfc_81_* [50]. Surface with high anisotropy and low complexity and heterogeneity of complexity characterize the species avoiding lignified tissues, but foraging mostly on monocots (grasses and sedges) and leaves. Browsing food, especially lignified tissues such as bark, buds and seeds, tends to lead to shearing molar facets with low anisotropy and high complexity [49,51,52].

### 2.3. Reconstruction of vegetation and climate

We used global vegetation for a series of 1 ka time slices [43] simulated by the LPJ-GUESS dynamic global vegetation model (DGVM) [53]. To obtain vegetation structure (the palaeobiome predictions) for sites in the Late Pleistocene, we extracted vegetation biomass (carbon mass) and leaf area index (LAI) for 11 plant functional types (PFTs) available for each 0.5° grid cell from the published dataset [43] using the geographic coordinates of bison specimens, and their calibrated radiocarbon ages. For the same geographic coordinates of radiocarbon-dated bison, climatic parameters (mean annual precipitation and mean annual temperature) were obtained from a statistics-based reconstruction of high-resolution global terrestrial climate [54]. For our study, data were generated using the NNjoin function in QGIS v. 3.20.0 (electronic supplementary material, S1). Vegetation reconstruction was carried out for 20 radiocarbon-dated specimens of *B. priscus* and for 1 specimen of Bb1 bison. LAI of the same vegetation types was summed to create three categories: tree, shrub and C3 grass for statistical comparison of vegetation between sites. Late Pleistocene biome types occurring at different sites were recognized based on carbon mass and LAI for the 11 PFTs by implementing the key in the appendices of the article by Allen *et al*. [43].

### 2.4. Statistical analysis

Using the dataset including all 157 specimens of bison from the 19 sites (figure 1), all DMTA parameters were transformed using the Box–Cox transformation to obtain a normal distribution of the variables [55]. Brown–Forsythe and Levene tests were performed to test for homogeneity of variance. One-way ANOVA was then used to test for differences between seven steppe bison populations (six sites with *n ≥ *10 individuals and one population called Yakutia—other grouped from individual specimens from different locations in Yakutia) and the Bb1 bison population. When ANOVA showed statistically significant differences, *post hoc* comparisons were performed using the conservative Tukey’s HSD test and the less conservative Fisher’s LSD test.

Also, we conducted AVOVA to identify differences in the reconstructed tree, grass and shrub LAI between sites with radiocarbon-dated individuals. All ANOVAs were carried out using Statistica (v. 9.1) [56].

To investigate factors influencing dental wear parameters in steppe bison, we ran separate models for each of the four parameters: complexity (*Asfc*), anisotropy (*epLsar*) and heterogeneity of complexity (*HAsfc*_*9*_ and *HAsfc*_*81*_) using 20 specimens with radiocarbon age (electronic supplementary material, S3). Due to the high correlation (*R* > 0.5), from the total set of available explanatory variables, LAI of tree, shrub and C3 grass, ^14^C age, mean annual temperature, mean annual precipitation, longitude and latitude, we excluded longitude (highly correlated with C3 grass LAI) and latitude (highly correlated with tree LAI), ^14^C age (highly correlated with C3 grass LAI), mean annual temperature (highly correlated with C3 grass LAI) and mean annual precipitation (highly correlated with tree LAI). The final model had three explanatory variables: tree LAI, shrub LAI and C3 grass LAI. It is worth noting that the LAI tree and LAI shrub here include data from both boreal and temperate species. We ran multiple linear regression models with each texture parameter as the response variable. The Akaike information criterion (AIC) with the second-order correction for a small sample size (AICc) was used for model ranking. If we did not find a single best model within any of the models run, we applied model averaging, where the cumulative weights of subsets of models did not exceed 0.95. We considered full model averaging to identify factors that significantly affect the DMTA parameters. Normality and homoscedasticity in the distribution of final model residuals were tested by inspecting the quantile–quantile distribution plot and the model residuals versus fitted values (estimated responses) plot. Multiple regression models were completed in R (v. 4.2.0) and RStudio (v. 2023.06.0) [57,58]. Model ranking was performed using the MuMIn package [59].

## 3. Results

### 3.1. Patterns of the diet

**Figure 2.**
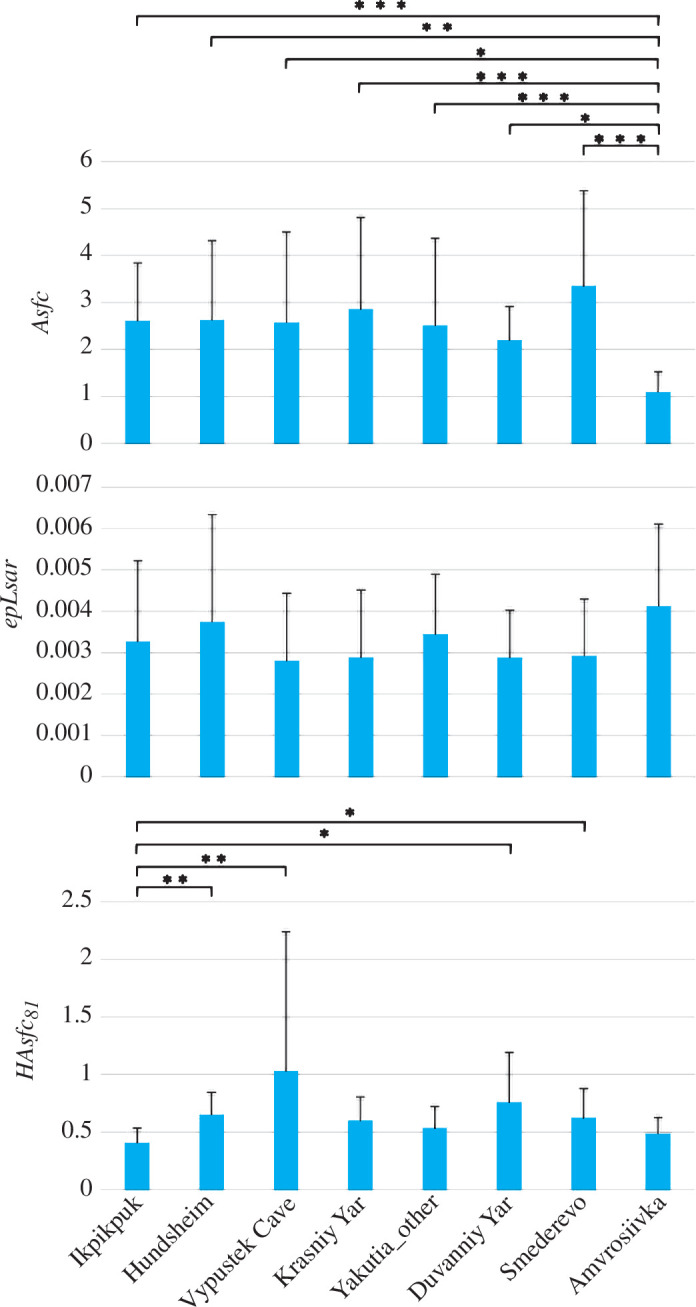
Differences in dental microwear texture parameters (complexity*—Asfc*; anisotropy*—epLsar*; and heterogeneity of complexity*—HAsfc_81_*) between the populations of the steppe bison *B. priscus* and Bb1 bison (Amvrosiivka) (statistically significant differences: *0.05 > *p* > 0.01; **p < 0.01; ***p < 0.001). Boxes indicate the mean and the whiskers indicate the standard deviation.

Eurasian and Alaskan steppe bison (*B. priscus*) populations did not show any significant differences in complexity (*Asfc* from 2.2 ± 0.7 in Duvanniy Yar, Yakutia to 3.3 ± 2.0 in Smederevo, Serbia), anisotropy (*epLsar* from 0.0028 ± 0.0017 in Výpustek Cave, Czech Republic to 0.0037 ± 0.0026 in Hundsheim, Austria—Middle Pleistocene) and heterogeneity of complexity with 9 cells (*HAsfc_9_* from 0.2 ± 0.1 in Ikpikpuk, Alaska to 0.4 ± 0.3 in Výpustek Cave) (figure 2; electronic supplementary material, tables S4.1 and S4.2).

**Figure 3.**
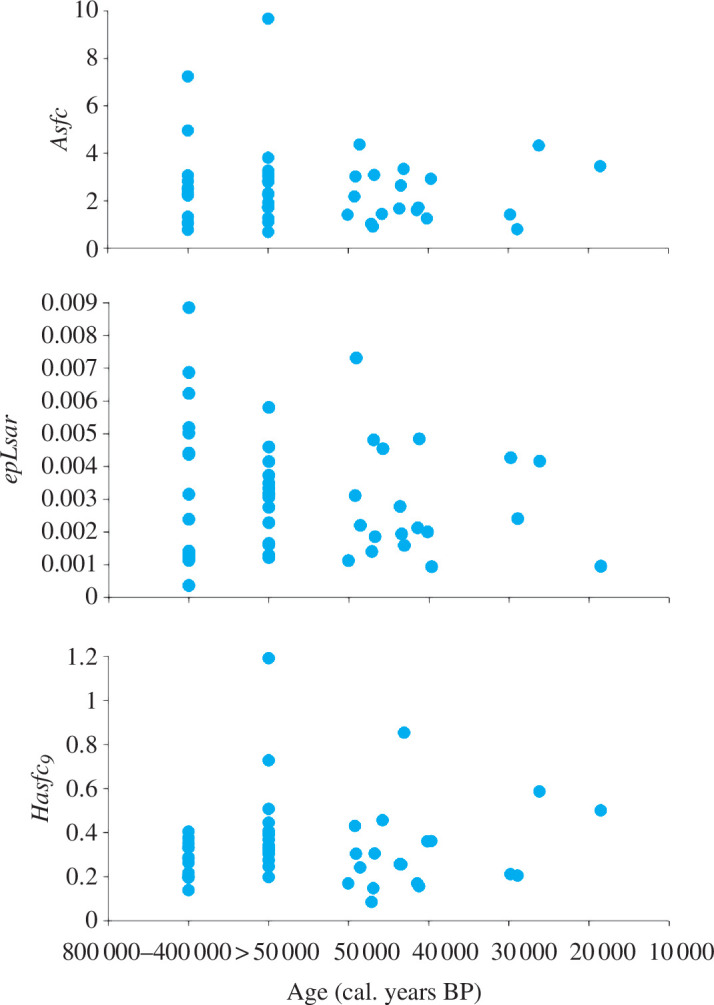
Dental microwear texture parameters of the steppe bison *B. priscus* over time in Eurasia and Alaska.

However, we found differences in *HAsfc_81_* between the population from Alaska (0.4 ± 0.1 the lowest mean) and three European populations: Hundsheim (0.7 ± 0.2), Smederevo (0.6 ± 0.2) and Výpustek cave (1.0 ± 1.2 the highest mean) and one population from Siberia: Duvanniy Yar (0.8 ± 0.4) (figure 2; electronic supplementary material, tables S4.1 and S4.2). Dental microwear textures of the Eurasian and Alaskan steppe bison did not vary significantly in time from the Middle Pleistocene onward (complexity*—Asfc*, *p* = 0.6; anisotropy*—epLsar*, *p* = 0.21; and heterogeneity of complexity*—HAsfc_9_*, *p* = 0.57) (figure 3).

Bb1 bison from the Amvrosiivka (Ukraine) was characterized by the highest mean anisotropy (*epLsar*)—0.0041 ± 0.002, and the lowest mean complexity (*Asfc*)—1.1 ± 0.4, among all bison populations (electronic supplementary material, table S4.1). Complexity (*Asfc*) significantly separated Bb1 from the steppe bison. One-way ANOVA revealed that Bb1 bison from Amvrosiivka differed significantly in complexity (*Asfc*) from all steppe bison populations (figure 2; electronic supplementary material, table S4.2).

### 3.2. Palaeoreconstruction of vegetation

Analysis of vegetation reconstruction has identified four types of biomes in the study area during the Late Pleistocene: temperate summergreen forest, steppe/temperate grassland, tundra and boreal broad-leaved summergreen forest. Steppe bison from Smederevo occupied the temperate summergreen forest and steppe/temperate grassland biomes, steppe bison from Western Siberia and Eastern Siberia occupied tundra-like biomes, while Bb1 bison from Amvrosiivka occupied the mosaic of boreal broad-leaved summergreen forest and steppe/temperate grassland biomes (electronic supplementary material, figure S4.1). According to the ^14^C dating, the youngest Late Pleistocene individual of *B. priscus* in this study, dated to 18 519 ± 224 cal. years BP (ETH-93367), was found in the Duvanniy Yar population (electronic supplementary material, figure S4.1). Most of the steppe bison were between 40 000 and 50 000 cal. years BP or older (refer electronic supplementary material, S1). Bb1 bison from Amvrosiivka was dated to 22 293 ± 153 cal. years BP (ETH-62961) (electronic supplementary material, figure S4.1).

Individual sites differed significantly in tree and grass cover, while shrub cover was similar in all analysed localities (electronic supplementary material, tables S4.3 and S4.4 and figure S4.2). All sites occupied by steppe bison had significantly lower grass cover than Amvrosiivka (Bb1). Smederevo and Amvrosiivka (Central–Eastern Europe) had similar and the highest tree cover (electronic supplementary material, tables S4.3 and S4.4 and figure S4.2). Despite significant differences in tree and grass cover between sites, the different populations of *B. priscus* show no significant variation in dental microwear texture parameters (figure 2; electronic supplementary material, tables S4.1 and S4.2).

**Figure 4.**
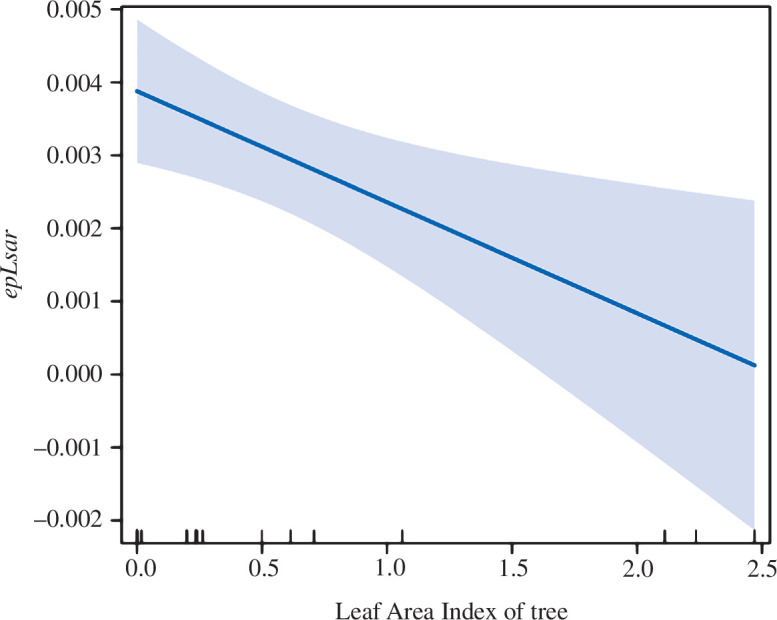
Predicted association between steppe bison tooth anisotropy (*epLsar*) and tree LAI in study sites. Shaded areas indicate 95% confidence intervals.

The results of the examined models indicated that the tree LAI decreased significantly with an increasing anisotropy (*epLsar*) of steppe bison teeth (LM: *p* = 0.0278, *R^2^* = 0.35; figure 4; electronic supplementary material, tables S4.5 and S4.6). The variation of the other tooth parameters (*Asfc*, *HAsfc_9_* and *HAsfc_81_*) was not explained by any of the factors considered (electronic supplementary material, tables S4.5 and S4.6).

## 4. Discussion

According to the DMTA, *B. priscus* was neither a grazer like modern cattle *Bos taurus* from the Camargue (France) [60,61] or semi-wild, unfed Heck cattle from Oostvaardersplassen (The Netherlands) [41] nor a browser like moose [52], but a mixed feeder. This is particularly evident in the variation in both complexity and anisotropy between samples. Similar to all Eurasian *B. priscus* populations, the Alaska (Ikpikpuk) Pleistocene *B. priscus* had a mixed diet. Complexity and anisotropy values of Alaskan steppe bison fall between modern American bison classified as obligate/variable grazers (*Bison bison bison* and *Bison bison athabascae*) [44] and the more browse-enriched mixed feeder European bison (*B. bonasus*) (electronic supplementary material, table S4.1) [41]. However, with the lowest *HAsfc_81_*, the Ikpikpuk population probably had a less variable diet than the European populations from Hundsheim, Smederevo and Výpustek Cave and the population from Siberia (Duvanniy Yar). Such a low *HAsfc_81_* as in the Alaskan population was observed in modern European bison from the Białowieża Forest (electronic supplementary material, table S4.1) [41]. In addition, mean stable nitrogen isotope signatures for five steppe bison from Ikpikpuk population (4.3‰) dated between ~14 215 and 44 930 cal. years BP confirm that the diet of Alaskan bison was mixed [42]. Similar nitrogen isotopic values (4.0‰) were found in the modern European bison population from the Knyszyn Forest in northeastern Poland, where they use coniferous forests and farmland [62].

The diet of the steppe bison probably included a wide range of vegetation types, as confirmed by several studies of very few singular frozen mummy specimens. DNA-based study of pollen samples from the colon of the two Yakutian steppe bison from permafrost dated to the beginning of the Holocene (about 10 500 cal. years BP) confirmed that the spring/summer diet was not strictly grazing. Apart from 71% of Poaceae pollen (grasses), it also contained herbs, shrubs (such as *Betula* sp. (birch) and *Salix* sp. (willow)) and 3–4% tree species (such as *Abies* sp., *Pinus* sp. and *Alnus* sp.), and came from a marshy wetland environment (marsh, bog, fen and swamp) [16]. According to morphological analysis of plant fragments in faecal samples from the same frozen bison mummies, the dominance of Poaceae and Cyperaceae in the bison diet was even lower—only 50% of vegetative remains, with the remainder being wetland forbs (e.g. *Comarum palustre* and *Menyanthes trifoliata*), as well as *Salix* sp. and minor moss fragments [15]. The plant macrofossil assemblage from inside the skull of the Alaskan steppe bison from Ikpikpuk (49 070 ± 2974 cal. years BP, UAMES 29458) included seeds of Poaceae and Carex (sedges), but also leaves of *Salix* spp., *Betula* spp. and *Andromeda polifolia* (bog rosemary)—shrubs typical of wet tundra—and seeds of Draba, *Papaver* sp., and Asteraceae—forbs of open tundra [63].

The mixed diet of the steppe bison examined by DMTA is similar to that observed in smaller modern bovid species such as chamois *Rupicapra rupicapra* or mouflon *Ovis gmelini musimon* from the Bauges Massif Regional Nature Park, France [64], where grasses represent less than 40–50% of their diet [65]. Although these bovids are highly hypsodont (a phenotype assumed to be an adaptation for grazing), they are not grazers but mixed feeders. Obligate grazing behaviour requires an abundance of rich grassland vegetation throughout the year. Such conditions exist in wetlands, where the effects of seasonal variation are buffered. In modern ecosystems in Africa, where drought severely affects grass (versus bush) biomass, grazers are either floodplain and aquatic marsh dwellers such as kob antelope (e.g. *Kobus leche*; Reduncini) [49] or migratory species such as wildebeest (e.g. *Connochaetes taurinus*; Alcelaphini) [66].

We would have expected habitat (and thus primary resource) variation to have influenced the diet of the steppe bison, as observed in other modern species such as red deer [67] or the Neolithic (6500–3500 cal. years BP) European bison (*B. bonasus*). The latter was more engaged in grazing in open habitats (sub-alpine meadows, Wetzikon-Robenhausen, Switzerland) than in densely forested regions of northeastern Europe [40]. However, despite the between-site differences in vegetation structure and biome type, all steppe bison populations from Eurasia and Alaska do not differ from each other when dental microwear textures were considered. In a gradient of decreasing tree cover from western Eurasia (Smederevo, Serbia), to eastern Siberia (Yakutia) and Alaska, all the steppe bison populations had mixed diets, which indicates a wide range of consumed items, but also a lower plasticity compared with modern European bison observed in the Holocene and today [40,62]. A first look at the sites for which we have vegetation reconstructions suggests that the (boreal/temperate) shrub component may account for these population-level foraging similarities, as it was the only vegetation component that did not show any significant variation in abundance between sites.

When looking at the factors that may have influenced the dental microwear textures of steppe bison (and so the diet), there is a negative correlation between dental microwear anisotropy and the proportion of tree vegetation (tree LAI). Although based on 19 individuals, such a negative correlation with tree cover actually reflects feeding on lower vegetation layers, including herbs and shrubs, whose biomass is inversely correlated with tree cover. Tree LAI was collinear with latitude and to the mean annual precipitation and was therefore excluded from the model. However, the importance of these two factors in explaining variation in dental microwear texture anisotropy is as high as that of tree LAI.

The present palaeoenvironmental reconstruction, performed individually for each radiocarbon-dated steppe bison specimen, together with other recent studies from Yakutia [16,68] undermine the common conviction that the Late Pleistocene *B. priscus* lived generally in a steppe habitat. Among the 19 directly radiocarbon-dated steppe bison individuals, 79% occupied the tundra biome, where herbaceous dicots and dwarf tree species are indeed abundant. The abundance of shrubs in the Late Pleistocene tundra was reflected in the tooth microwear of this large bovid. The prevalence of a Late Quaternary graminoid-dominated Arctic ‘mammoth steppe’, as well as obligate grazing by large herbivores during this period, has already been questioned by a large-scale ancient DNA metabarcoding study of circumpolar plant diversity [69]. Similarly, the taxonomic composition of the plant remains found in the gastrointestinal tract of the frozen carcass of a Pleistocene bison dated to 35 693–31 415 cal. years BP from Mylakhchin in Yakutia suggests that the species occupied a specific ecological niche, which could have been provided by a biome similar to modern taiga, Arctic heathland and shrubland with dwarf birch *Betula nana*, but not by the steppe habitat [68]. This study suggests that similar to bison, the diets of reindeer, horses and most mammoths and rhinoceroses corresponded more to tundra, forest and bog than to steppe vegetation over time, including the Last Glacial Maximum and periods before and after it, suggesting that megaherbivores avoided dry steppe as a pasture [68].

DMTAs allowed us to examine, for the first time, detailed dietary patterns of Bb1 bison (called also Clade X)—a recently genetically identified clade of the genus *Bison* that lived in Europe during the Late Pleistocene and became extinct in the early Holocene [25]. The diet of Bb1 bison, at least in Amvrosiivka, was typically grassy, despite a higher proportion of trees than grasses available in the environment. The diet of Bb1 clearly distinguishes this genetic clade from all of the *B. priscus* populations in Eurasia and Alaska. Although the bone bed at Amvrosiivka probably resulted from numerous hunts and subsequent butchering [70], the low intra-population variation in complexity suggests that the bison could have been captured and killed in large numbers over a short period of time. Hunting over several months or seasons would have revealed higher intra-population variation in samples, as food resources vary in composition and quality from one season to another [71,72]. In contrast to Bb1 bison, the wider range of values for dental microwear texture parameters in different *B. priscus* populations supports possible death events over a longer period of time and most probably reflects annual dietary variation. Thus, we suspect that the DMTA results of Bb1 reflect seasonal windows that correspond to the peak of herbaceous monocot abundance in the environment. Alternatively, Bb1 could be depicted as an obligate grazer all over the seasons. It is therefore essential to deepen this preliminary study of the diet of the Bb1 bison at larger spatial and temporal scales, and to extend it to other populations that have already been identified in many different locations in Europe [73].

Data from Siberia indicated that the extinction of megafauna in the north of Eastern Siberia was significantly delayed compared with other regions of Eurasia [74]. This early extinction in Europe in the case of the last mammoths is explained by the rapid spread of the boreal forest and the loss of the steppe–tundra biome [75]. A possible explanation for the longest survival of the steppe bison in Yakutia comes from a multi-species modelling study, which suggests that landscape fragmentation played a major role in the extinction of megafauna in Eurasia [2]. Although modelling experiments provided little evidence for long-term biome stability on a global scale [43], the preliminary vegetation reconstruction we proposed at the steppe bison occurrence sites in Yakutia showed that the tundra subsisted from at least 48.5 to 18.5 ka BP. The possible role of humans in the extinction of the Late Pleistocene megafauna is also considered. According to the recent studies, the extinct species begun their path to extinction by losing occupied territories and splitting into increasingly isolated patches almost simultaneously with the arrival of anatomically modern humans in Eurasia [76,77]. Other pattern-oriented modelling studies also found that climate change and human hunting interacted to cause the extinction of the steppe bison in its last refuge in Siberia. It underlies that since the Last Glacial Maximum, hunting alone could not have driven the steppe bison to extinction meaning that without climate warming negatively affecting range and abundance, the steppe bison were projected to be at large abundances at 5000 years BP despite human hunting [78]. Therefore, we might conclude that environmental factors, such as the long-term stability of the biome in Yakutia, have played an important role in the species’ prolonged survival in this region despite the anthropogenic impact.

## 5. Conclusions

*Bison priscus* was not a specialized grazer, but a mixed feeder in a large gradient of different biomes that occurred in Eurasia and Alaska during the Late Pleistocene. We confirmed that despite their adaptation to grazing, steppe bison included easily digestible non-grass vegetation in their diet, similar to modern bison species—American and European bison [79,80]. Steppe bison might have survived the longest in Eastern Siberia due to the stability of the tundra biome over tens of thousands of years in this region. However, neither the steppe bison, with its broad mixed diet, nor the Bb1 bison, with its potentially highly specialized grass diet, has managed to avoid extinction. Even the habitat stability of Eastern Siberia did not ensure the survival of the steppe bison. Numerous factors other than feeding ecology, including the specific effect of climate warming on large-body-size mammals, which were not examined in this study, may have had a significant effect on the species’ inability to adjust to the changing environmental and climatic conditions at the turn of the Pleistocene and Holocene periods.

## Data Availability

Data and codes are available in the electronic supplementary material [81].
